# Financial difficulties but not other types of recent negative life events show strong interactions with 5-HTTLPR genotype in the development of depressive symptoms

**DOI:** 10.1038/tp.2016.57

**Published:** 2016-05-03

**Authors:** X Gonda, N Eszlari, D Kovacs, I M Anderson, J F W Deakin, G Juhasz, G Bagdy

**Affiliations:** 1 Department of Pharmacodynamics, Faculty of Pharmacy, Semmelweis University, Budapest, Hungary; 2 MTA-SE Neuropsychopharmacology and Neurochemistry Research Group, Hungarian Academy of Sciences, Semmelweis University, Budapest, Hungary; 3 Department of Psychiatry and Psychotherapy, Kutvolgyi Clinical Center, Semmelweis University, Budapest, Hungary; 4 Neuroscience and Psychiatry Unit, Institute of Brain, Behaviour and Mental Health, Faculty of Medical and Human Sciences, The University of Manchester, Manchester, UK; 5 Manchester Academic Health Sciences Centre, Manchester, UK; 6 MTA-SE-NAP B Genetic Brain Imaging Migraine Research Group, Hungarian Academy of Sciences, Semmelweis University, Budapest, Hungary

## Abstract

Several studies indicate that *5-HTTLPR* mediates the effect of childhood adversity in the development of depression, while results are contradictory for recent negative life events. For childhood adversity the interaction with genotype is strongest for sexual abuse, but not for other types of childhood maltreatment; however, possible interactions with specific recent life events have not been investigated separately. The aim of our study was to investigate the effect of four distinct types of recent life events in the development of depressive symptoms in a large community sample. Interaction between different types of recent life events measured by the List of Threatening Experiences and the *5-HTTLPR* genotype on current depression measured by the depression subscale and additional items of the Brief Symptom Inventory was investigated in 2588 subjects in Manchester and Budapest. Only a nominal interaction was found between life events overall and *5-HTTLPR* on depression, which failed to survive correction for multiple testing. However, subcategorising life events into four categories showed a robust interaction between financial difficulties and the *5-HTTLPR* genotype, and a weaker interaction in the case of illness/injury. No interaction effect for the other two life event categories was present. We investigated a general non-representative sample in a cross-sectional approach. Depressive symptoms and life event evaluations were self-reported. The *5-HTTLPR* polymorphism showed a differential interaction pattern with different types of recent life events, with the strongest interaction effects of financial difficulties on depressive symptoms. This specificity of interaction with only particular types of life events may help to explain previous contradictory findings.

## Introduction

Genetic variation in the serotonin transporter is one of the most widely studied risk factors for depression since the original observation of its association with neuroticism and interaction with life events.^1, 2^ However, its precise role in the development of depression and in mediating the effects of life events is still controversial in spite of a large number of studies.

*5-HTTLPR* is a functional repeat length polymorphism influencing serotonin reuptake efficiency and consequently serotonin availability in the synaptic cleft in the central nervous system. The short (s) allele, associated with reduced efficiency of transcription of the serotonin transporter protein, has been found to predispose to various depression-related constructs^2, 3, 4, 5, 6, 7, 8^ and depression itself^9, 10^ in several human and animal studies.^11^ An initial large-scale follow-up study by Caspi *et al.* indicated a key role for the s allele of *5-HTTLPR* in mediating the effects of stressful life events in the development of depression.^1^ This was followed by a number of contradictory replication studies and meta-analyses,^12, 13, 14, 15^ with the latest and most comprehensive meta-analysis once again supporting such an association.^16^ In the background of contradictory results, one of the more commonly proposed reasons was the lack of stratification according to the timing of negative life events. When categorised into early childhood and recent life events, an association between the *5-HTTLPR* s allele and early-life adversities, but not recent life events, was found with depression.^15, 16, 17, 18, 19, 20^ Early-childhood adverse experience is proposed to exert a long-lasting impact on neurodevelopment through influencing serotonergic neurotransmission in neural circuits responsible for mood regulation. This increases the vulnerability to the negative effects of early stressors,^21, 22, 23^ as well as causing other possible structural and functional changes, including alterations in the programming of glucocorticoid, noradrenergic and vasopressinergic stress response systems. As a result these systems are hypothesised to show a long-lasting over-reaction to new stressors, leading to significantly impaired hippocampus and amygdala development.^24, 25, 26^

Less is known about the neurobiological mechanisms by which the *5-HTTLPR* mediates the effects of recent life events and acute stressors in increasing the risk of the development of psychopathology.^27^ It has been suggested that presence of the s allele increases stress sensitivity by biasing those neurobiological systems that underlie threat reactivity and arousal.^27^ An association between processing images of fearful and negative expressions, but not positive expressions, and increased amygdala reactivity and *5-HTTLPR* genotype has been found in several studies.^28, 29^ Furthermore, during acute stress exposure, ss carriers preferentially modulate their attention towards threat, perseverate on the emotional salience of threat, and exhibit a tendency for preferential engagement of fear- and arousal-enhancing neural systems due to an enhanced activation of a neural network including the amygdala, hippocampus, anterior insula, thalamus, pulvinar, nucleus caudatus, precuneus, anterior cingulate cortex, and the medial prefrontal cortex.^27^ A recent meta-analysis also found a small but significant association between *5-HTTLPR* genotype and hypothalamic-pituitary-adrenal-axis reactivity during acute psychosocial stress, with a higher cortisol response in those carrying the s allele, potentially contributing to a heightened risk for the development of stress-related disorders.^30^

The type of childhood adverse event appears important, with most studies indicating a robust interaction between *5-HTTLPR* genotype and early sexual abuse, but not other types of childhood maltreatment, in the development of adult depression.^17, 31, 32^ These observations suggest that different types of stressors and life events may act via differential biological pathways. However, less attention has been paid to the categorisation of recent life events, although the marginal association seen in the largest meta-analysis^16^ and the results of several individual studies reporting an interaction between recent life events and *5-HTTLPR*^33, 34, 35, 36^ point to an aetiological role of this interaction in the development of depression. This raises the possibility that stratification according to different types of recent life events may reveal important mechanisms concerning the role of *5-HTTLPR*. However, large-scale studies focusing on separating different types of recent life events are lacking. It is also noteworthy that a study found that recent life events specifically related to financial hardships interact with neural nitric oxide synthase polymorphisms in the development of depression, indicating a prominent role for this type of life events in the appearance of depression.^37^ Therefore, the aim of the present study was to investigate the contribution of different types of recent life events (RLE), related to intimate relationships; financial difficulties; illnesses and injuries; and social network problems to the interaction with the *5-HTTLPR* polymorphism in the emergence of depressive symptoms in a large community population recruited from Manchester and Budapest.

## Materials and methods

### Population

Volunteers aged between 18 and 60 years have been recruited through general practices, advertisements and a website (http://www.newmood.co.uk) from Greater Manchester, UK and Budapest, Hungary, under the aegis of the NewMood study (New Molecules in Mood Disorders, Sixth Framework Program of the European Union LHSM-CT-2004-503474). A questionnaire pack and a genetic sampling kit were sent to each participant who signed the official informed consent form provided by mail. From the total recruited 2588 participants we carried out our analysis in a group of 2234 subjects (896 from Budapest and 1338 from Manchester). We included only non-related individuals with European white ethnic origin who could be genotyped for the *5-HTTLPR* polymorphism and provided eligible phenotypic data. More details about the population sample can be found in our previously published reports.^36, 38, 39^ This study was conducted in accordance with the Declaration of Helsinki, and has been approved by the local ethic committees (North Manchester Local Research Ethics Committee, Manchester, UK; Scientific and Research Ethics Committee of the Medical Research Council, Budapest, Hungary). All subjects gave written informed consent before participating in the study.

### Phenotypic data

We used the calculated continuous weighted dimension score of the Brief Symptom Inventory depression subscale with the addition of the additional four items in order to assess the current depression state.^40^ Recent life stress (RLE) was measured by the List of Threatening Experiences questionnaire^41^ divided into four validated subscales (Supplementary Table S3), and covering the events occurring in the previous 12 months. The four subscales included intimate relationship problems (RLE-relationship), financial difficulties (RLE-financial), illness/injury (RLE-illness) and social network disturbances (RLE-social).

### Genetic data

DNA extraction was carried out on self-provided buccal mucosa cells of the subjects by validated methods.^42^ The *5-HTTLPR* genotype was determined as described elsewhere.^36^ All laboratory work was performed under the ISO 9001:2000 quality-management requirements and was blinded with regard to phenotype.

### Statistical analysis

The Quanto programme (http://biostats.usc.edu/Quanto.html) was used for power calculations before the main analysis. The statistical power of the analysis is enough to find the main effect of the *5-HTTLPR* polymorphism with an assumed *R*^2^=1% explained variance in an additive heritability model by 99% probability. In addition, it has 99% power to find interaction between continuous interacting environmental effect RLE and *5-HTTLPR* with assumed additive heritability and *R*^2^=1% explained variance.

The PLINK programme (http://pngu.mgh.harvard.edu/purcell/plink) was used to determine Hardy–Weinberg equilibrium, and for genetic association analyses. Additive, dominant and recessive models were all tested with age and gender as covariates in all models. In case of interactional models, the main effect of the genotype and that of the life event variable were also included as covariates. *Post hoc* tests of all models were carried out in the two genders to establish gender differences. Nominal significance threshold was *P*<0.05, and we calculated false discovery rates (FDR) for the main tests by using the QVALUE programme http://github.com/jdstorey/qvalue. As we have large samples (*n*>200), parametric statistical methods were applied based on the central limit theorem.^43^ SPSS 20.0 (IBM, Armonk, NY, USA) for Windows was used for other statistical analyses, and to generate the figures.

## Results

Descriptive data and statistics of our study population are presented in Table 1.

Our polymorphism was in Hardy–Weinberg equilibrium in both independent populations (Budapest *P*=0.79 and Manchester *P*=0.91) and in the combined sample (*P*=0.77). Pearson's correlations were calculated between the four subscales of the List of Threatening Experiences questionnaire in the combined sample, resulting in significant but negligible weak correlations with the exception for the correlation between RLE-relationship and RLE-social, as well as RLE-financial and RLE-social, which were nonsignificant (Supplementary Table S1).

### Main effect of the 5-HTTLPR polymorphism on current depressive symptoms

Main effect of the polymorphism was only detectable in the population sample of Budapest (896 subjects), using additive (*P*=0.0497) and recessive (*P*=0.0133) models. After calculating for FDRs with the QVALUE programme, the significant main effect of *5-HTTLPR* in the additive and recessive models no longer met the significance criteria (Tables 2, 3, 4).

*5-HTTLPR* had no significant effect on experiencing and/or reporting recent life events, either in whole or according to subcategories (Supplementary Table S2).

### Interaction between the 5-HTTLPR genotype and total RLE score on current depressive symptoms

Interaction with the total RLE score was nominally significant in the combined sample, using additive (*P*=0.0327) and recessive (*P*=0.0381) models. However, none of them passed the FDR test performed by the QVALUE programme (Table 2).

### Interaction between individual recent life event subscales and the 5-HTTLPR genotype on current depressive symptoms

**Figure 1:**
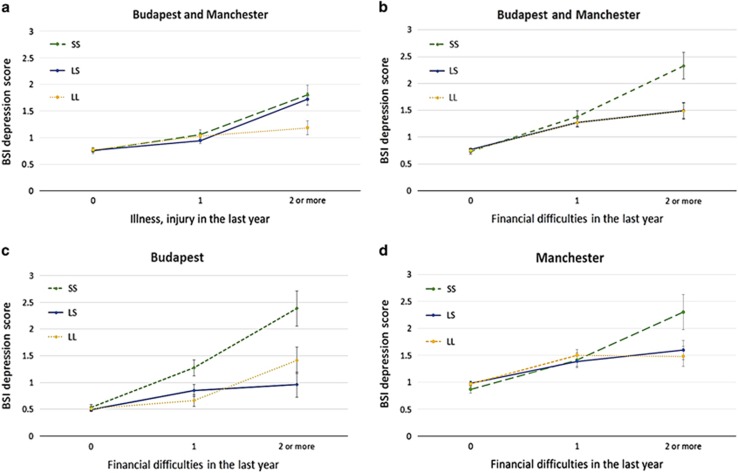
Linear regression analysis indicated a significant interaction effect between the *5-HTTLPR* genotype and specific types of recent life events on current depression scores in the total population and subpopulations. Recent life events are categorised as 0 (no recent life events reported), 1 (1 life event reported) and 2 or more life events reported in the last year. s.e.m. is displayed. Linear regression indicated a significant interaction between the *5-HTTLPR* genotype and recent life events related to illness and injury (RLE-illness) on current depression scores in the combined (Budapest+Manchester) sample according to the additive (*P*=0.016, FDR *q*=0.038) and dominant (*P*=0.009, FDR *q*=0.035) models (**a**); a significant interaction effect between the *5-HTTLPR* genotype and recent life events related to financial difficulties in the last year (RLE-financial) on current depression scores in the combined (Budapest+Manchester) sample according to additive (*P*=0.017, FDR *q*=0.038) and recessive models (*P*=0.002, FDR *q*=0.016) (**b**); a significant interaction effect between the *5-HTTLPR* genotype and recent life events related to financial difficulties in the last year (RLE-financial) on current depression scores in the Budapest sample according to additive (*P*=0.007, FDR *q*=0.035) and recessive models (*P*=0.001, FDR *q*=0.012) (**c**); and a significant interaction effect between the *5-HTTLPR* genotype and recent life events related to financial difficulties in the last year (RLE-financial) in the Manchester sample on current depression scores according to the recessive model (*P*=0.00997, FDR *q*=0.035) (**d**). The increasing number of s alleles increases current depression score with the increasing number of recent life events related to illness and injury (**a**) or financial difficulties according to the additive model (**b**, **c**), and current depression score increases with the increasing number of recent life events related to financial difficulties in those carrying the ss genotype according to the recessive model (**d**). FDR, false discovery rate.

Among RLE subscales (see item composition in Supplementary Table S3) only financial difficulties (RLE-financial) and illness/injury (RLE-illness) had a significant modulating effect in interaction with *5-HTTLPR* on the Brief Symptom Inventory depression phenotype (Tables 2–4). The number and frequency of subjects reporting 0, 1, 2 or 3 financial life events and illness/injury-related life events are shown in Supplementary Table S4. To visualise our significant interaction results, we plotted Brief Symptom Inventory depression scores in groups endorsing 0, 1 and 2 or more life events (Figure 1).

RLE-illness contains four questions about serious illness, injury or problems occurring between the subject and close relatives, neighbours or the police. In the combined sample using additive (*P*=0.0156) and dominant (*P*=0.0088) model of heritability significant interactions with *5-HTTLPR* were found, and these were not false discoveries according to the QVALUE programme (Table 2, Figure 1a). Independently in the Budapest sample using a dominant (*P*=0.0365) model we found a significant interaction; however, it did not survive the test for FDRs (Table 3) indicating no replication of our result in the two population subsamples.

RLE-financial is a three-question subscale of RLE with items about financial difficulties, unsuccessful attempts to find a job or being fired. In all three population samples (Budapest, Manchester and combined) association of the RLE-financial score in interaction with the *5-HTTLPR* polymorphism was significant by using additive or recessive model of heritability. These significances survived the test for false discoveries with one exception in the Manchester sample (additive model *P*=0.0481) showing the replicability of the result in the two population subsamples. *P*-values of the significant interactions are presented in Tables 2, 3 and 4 and Figures 1b, c and d.

### *Post hoc* tests of the interaction effects of life events in the two genders

After carrying out *post hoc* tests separately in the two genders for all interaction effects and the main effect of *5-HTTLPR*, all significant effects disappeared in women in the combined sample (Supplementary Table S5). In case of men (Supplementary Table S6), there was a significant interaction effect between *5-HTTLPR* and total life events using additive (*P*=0.0014) and recessive models (*P*=0.0001), which could be replicated both in the Budapest (additive model: *P*=0.0151, recessive model: *P*=0.0001) and Manchester subsamples (additive model: *P*=0.0201, recessive model: *P*=0.0322). Furthermore, in men we also found a significant interaction effect of RLE-financial in the combined sample (additive model: *P*=0.0010, recessive model: *P<*0.0001), which could be replicated in the two independent subsamples of Manchester (additive: *P*=0.0418, recessive: *P*=0.0076) and Budapest (additive: *P*=0.0013, recessive: *P<*0.0001). The other results could not be replicated in our study.

## Discussion

In our study we detected a robust significant interaction effect between the *5-HTTLPR* genotype and recent life events specifically and selectively related to financial difficulties (RLE-financial) in a combined sample from Manchester and Budapest. The interaction could be replicated both in the Budapest and Manchester samples when investigated separately. We also demonstrated a less marked interaction between life events related to illnesses and injury (RLE-illness) and *5-HTTLPR* genotype, which was only observable in the combined sample according to the additive and dominant models. Two other types of life events (those related to intimate relationships and those related to social network problems) showed no interaction effect with the *5-HTTLPR* genotype on the emergence of depressive symptomatology in the combined sample or any of the two subsamples. Our results, therefore, indicate a specific and selective effect for certain types of recent life events, namely those associated with financial difficulties, and less robustly illness/injury, in interaction with the *5-HTTLPR* genotype to influence the emergence of depressive symptomatology. The findings corroborate earlier findings concerning the role of the *5-HTTLPR* genotype in depression in modulating the effects of negative life events,^1, 12^ and have also potentially thrown light on the contradictory findings of several studies and meta-analyses,^16^ by showing that different types of recent life events and stresses may have a differential role in the emergence of depression, with only the effect of certain types of life events being modulated by *5-HTTLPR*.

### Selective effects of recent life events related to financial difficulties in interaction with the 5-HTTLPR genotype in the development of depressive symptoms

Our studies indicate a robust and selective effect for financial-related life events in ss genotype carriers with a weaker effect for illness and injury-related life events. We detected no or negligible correlation between life events related to financial problems and those related to illness/injury, suggesting independent effects. In addition, in our present study the *5-HTTLPR* genotype had no significant effect on the occurrence of any type of life stressors, therefore, the significant interaction effects we observed were not due to the increased number of the given type of RLEs in s carriers.

Our findings are not only novel but could also help to explain why contradictory results emerged in previous studies not stratifying for different types of recent life events. Furthermore, our findings are similar to those reported in case of childhood adversities where it was also demonstrated that not all types of early childhood maltreatment, but most prominently childhood sexual and to a lesser extent physical abuse is modulated by *5-HTTLPR*.^17, 31, 32, 44^ These results suggest that different types of life events are possibly modulated by different neural mechanisms involving different neuroanatomical structures and neurochemical components, and different genes. It is increasingly suggested that the environmental context related to stressful and adverse events should be specified more accurately and more multidimensionally because stress and adverse events are very broad terms. They encompass a wide variety of environmental effects and contexts that may have divergent consequences. Identifying which environments and effects are most prominent in such genetic interactions would help to identify those who are at greatest risk.^45^ In spite of this, apart from a previous study in adults reporting that the *5-HTTLPR*-rs25531 mini-haplotype moderates the relationship between depression and separation from partner within the preceding year in a synergistic manner,^46^ the effect of specific types of life events has been little studied; therefore, we have a limited understanding of what neurochemical and neuroanatomical mechanisms may modulate any differential effect.

Imaging studies support the well-known difference between early and recent life events in the interaction with *5-HTTLPR* and also provide evidence that both influence central nervous system activation in regions important in depression.^47^ Stressful recent life events, measured by a self-report questionnaire, were found to interact with *5-HTTLPR* genotype on hippocampus and amygdala resting-state activation, and an interaction effect on the functional connectivity of the hippocampus and the amygdala with a wide network of several brain regions and grey matter structural characteristics was also reported.^48^ Increased functional coupling between the right amygdala and the hypothalamus was found in response to fearful faces in s allele carriers and in ss genotype carriers exposed to a higher number of self-reported stressful life events measured by a self-report life events checklist.^49^ Stressful life events, measured by a structured interview based on a life-chart method, interacted with the *5-HTTLPR* genotype on the structural connectivity between the hippocampus and the amygdala as well as the putamen, and also on the functional connectivity of the parahippocampus with the posterior cingulate cortex.^50^ These studies provide a strong argument that recent life events in interaction with the *5-HTTLPR* genotype influence the activation patterns of brain areas implicated in depressive symptomatology, although the differential effects of types of recent life events have not been studied.

Previous work has supported the importance of financial stress in the development of depression. Finance-related and economic problems have been found to be related to the risk of onset and persistence of depressive episodes in previous studies.^51^ In a study investigating a broad range of risk factors predicting recurrence of depression in women, severe financial problems were the strongest predictors in the overall model^52^ and major financial crisis was among the most robust past-year stressful life event predictors of suicide attempts in 6004 major depressives.^53^ Furthermore, financial hardships, including having a lower income and being unemployed, were also associated with lower remission rates in depressed outpatients treated with citalopram in the STAR*D study,^54^ indicating a complex and multifaceted role of financial difficulties in contributing to the development of depression and having an impact on its course.

In studies investigating the contribution of genetic and environmental factors in the development of depression, several twin studies have reported a prominent effect for financial difficulties. For example, in monozygotic female twins discordant for major depression, eight maximally discriminating variables were identified, including current financial difficulties that predicted life history of major depressive disorder in a stepwise multivariate analysis.^55^ Income and financial status was found to predict early remission from major depressive episodes but not remission during the later course in a study in 1030 female twin pairs.^56^ Furthermore, when focusing on gender differences, financial, occupational and legal life events showed a stronger effect on the development of depressive episodes in males according to a study of 1057 opposite-sex dizygotic twin pairs.^57^

However, genetic liability to major depression may also increase the risk of being exposed to stressful life events, as it was found in one study investigating 2164 female twins where genetic liability for major depression was associated with a significantly increased risk for six types of personal stressful life events including major financial problems. This study concluded that women with a genetic predisposition for major depression may also be more prone to expose themselves to high-risk environments.^58^ A recent genome-wide association study also reported the heritability of reporting of recent life events pointing out that ~30% of the variance of self-reported environmental events can be explained by common genetic variants, although association analyses for specific single-nucleotide polymorphisms yielded only suggestive significance values.^59^ In our study, however, no significant effect of *5-HTTLPR* on life events was found; therefore, this is not responsible for the observed effects.

Financial problems and difficulties may encompass several effects related to economic and financial as well as social difficulties threatening the general safety and well-being of the individual and those dependent upon them. They may also be associated with partly gender-dependent role losses or injuries related to being the provider for the family, which may similarly contribute to excessive stress. Further possible mediating mechanisms may be that by their pervasive impact financial difficulties may contribute to such stresses as prolonged existential insecurity and feelings of loss of control. Recently, financial hardship in interaction with neural nitric oxide synthase polymorphisms was found to be related to depression,^37^ indicating a possibly specific role of these types of life events in the vulnerability or susceptibility for depression modulated by genetic polymorphisms. The majority of financial problem-related life events also include an immediacy of threat not captured in social network- or illness-related life events, which may also account for their closer interaction with *5-HTTLLPR* found to be related to threat sensitivity and threat reactivity.^27, 28^ Further supporting the pervasive impact of severe financial hardships, recently a study reported that childhood poverty was associated with reduced default mode network connectivity in adulthood, which latter was also associated with increased saliva cortisol in anticipation of social stress, indicating a possible neural basis for altered cognitive processing and exaggerated stress sensitivity in adults, which are the key aetiological factors in depression in those suffering from chronic poverty during childhood.^60^

### Interaction of life events and 5-HTTLPR in the two genders

Analysing the two genders separately, we found that all significant associations disappeared in women, whereas, in case of men, the interaction effect of the *5-HTTLPR* genotype with total life events as well as the interaction effect with financial-related life events was robustly significant in the combined sample and also replicable in the two subsamples.

Previous research indicates that *5-HTTLPR* variants exert their effect on a number of phenomena in a gender-based manner, and this is true also in interaction with environmental stressors. Although several studies did not consider gender or produced inconsistent results, a general pattern of findings indicates that the *5-HTTLPR* s allele has opposite effects in the two genders, increasing risk of depression in females but acting as a protective factor or having no effect in males, and the same pattern is observable also when interaction with stressful life events is considered.^61, 62, 63, 64^ A recent systematic review of 78 papers on gender differences in the effect of *5-HTTLPR* in affective spectrum disorders concluded that in interaction with stressful events presence of the s allele is associated with an increased risk of depressive symptoms, depression, trait or symptomatic anxiety and internalising behaviour in women, and with aggression, conduct disorder and externalising behaviours in men.^63^ Gender differences in the effects of *5-HTTLPR* were also reported in functional imaging^65^ and resting-state electroencephalogram studies.^66^ Specifically, greater synchronisation of regional neural organisation and the modulation of EEG activity in a wide frequency range was found in s-carrier women contributing to gender differences in cognition and emotion as well as affective states.^66^

Differences in the effect of *5-HTTLPR* are plausible, given that the serotonergic function shows gender differences including higher synthesis rate^67^ and 5-HT1A and 5-HT2 receptor densities^68, 69^ in males, and higher cerebrospinal fluid 5-HIAA concentrations,^70, 71, 72^ serotonin transporter availability^73^ and more severe depressive symptoms following tryptophan depletion^74, 75^ in females. The *5-HTTLPR* ss genotype was associated with higher cerebrospinal fluid 5-HIAA levels in females and lower levels in males,^71^ and, in response to tryptophan infusion, negative effects were more pronounced in s allele carrier females as well as l allele carrier males as opposed to other groups.^70^

It is likely that the gender effect observable in case of *5-HTTLPR* may be related to the effect of gonadal steroids influencing the expression of genes regulating serotonin synthesis and metabolism and removal of serotonin from the synapse and stimulation of pre- and postsynaptic serotonergic receptors.^76, 77, 78, 79, 80^ *5-HTTLPR* was also hypothesised to influence development of the serotonergic system in a gender-dependent manner,^81^ and, as the serotonergic system has a role in proliferation, migration, differentiation and synaptogenesis, establishment and function of brain circuits modulated by genetically based differences in serotonergic function may be different in men and women.^66^ It is also plausible that there are gender differences in responding with depressive symptoms to different stressors because different reproductive strategies are effective in the two genders.^61, 82, 83^

Therefore, our results contradict previous findings as they reflect an opposite pattern compared with what has been reported in previous studies, with the s allele in interaction with total life events or specifically with financial life events showing robust association with an increased depression score in males but not in females. It is possible that specifically financial stressors have a higher impact on men as they are related to pervasive loss of existential safety as well as of the traditional male role. However, the lack of interaction effect between *5-HTTLPR* and other types of life events (life events related to illness and injury, intimate relationships and social network) in women is a surprising new finding in the light of previous results.

### No significant main effect for 5-HTTLPR or in interaction with total life events in the development of depressive symptoms

It is also noteworthy that in our study we found no significant main effect for the *5-HTTLPR* genotype in the development of depressive symptoms in the combined sample from Budapest and Manchester, although a weak but significant association was detected only in the Budapest sample according to the recessive model. Our study thus supports the notion that *5-HTTLPR* is involved in the risk for the development of depression via its effect on mediating life events and not because of its direct effect on the emergence of depressive symptoms.

Furthermore, in accordance with several earlier studies and certain meta-analyses^13, 14^ our results have also shown that life events considered as a whole showed only a weak nominal interaction effect with *5-HTTLPR* in the development of depression, which did not survive correction for multiple testing in either population. In a previous study in a partially overlapping sample^33^ we also reported a weak interaction effect between the *5-HTTLPR* genotype and recent life events on lifetime depression and current depressive symptoms. These findings support a role for the *5-HTTLPR* genotype having a role in mediating the effects of recent life events, and a differential effect with respect to the type of event. This informs the debate as to whether all types of life events or only childhood adverse events are mediated by the *5-HTTLPR* genotype, and is in accordance with the results of the latest and largest meta-analysis,^16^ showing only a marginal association in case of recent life events.

### Limitations

Several limitations of our study must be noted. Our research was cross-sectional; thus, we could not evaluate the longitudinal effects of life events and depression was also measured cross-sectionally. Therefore, it is possible that depression developing in a longer time following very recent life events could not be identified in our study. In addition, we could not determine the timing of life events relative to depression. Furthermore, our study sample is a general, non-epidemiological and non-representative population sample based on volunteers and, therefore, may be subject to sampling bias, especially with respect to depression. In addition, all psychometric measurements including Brief Symptom Inventory and life events were based on self-report. We only subcategorised our life events into four, although validated, categories; it is therefore possible that some categories should be further refined. Although correlations between different categories of life events in the present study were weak, it is also possible that in isolated cases these life events are not independent of each other, which may influence the results.

### Conclusions and implications for further research

In conclusion, our findings indicate that in spite of previous contradictory results the *5-HTTLPR* genotype shows a strong interaction but only with selected types of life events, most robustly with life events related to financial difficulties and to a lesser extent life events related to illnesses and injuries in the development of depressive symptoms, although no such interaction was found between *5-HTTLPR* genotype and life events related to intimate relationships or to social network problems. These results are notable, given that we found only a weak main effect for the *5-HTTLPR* genotype on depression and a weak interaction effect that did not survive correction when life events were not separated according to type. Therefore, our results strongly argue for the mediating effect of *5-HTTLPR* between certain types of life events and depression, and may help to elucidate why previous studies yielded conflicting results. Understanding the differential effects of different types of stressors and adverse events, and how these may be mediated by different genetic components in the development of depression would be crucial to identify novel targets for early prevention, screening and intervention as well, both on the psychotherapeutic and pharmacotherapeutic levels.

## Figures and Tables

**Table 1:** Descriptive statistics of the measured phenotypes

*Gender*	*Male*		*Female*	
	685		1593	
				
	*Minimum*	*Maximum*	*Mean*	*s.d.*
Age	18	60	32.79	10.48
BSI depression score	0	4	0.8638	0.9272
Recent life events	0	8	1.23	1.299
RLE-relationship	0	2	0.13	0.377
RLE-financial	0	3	0.22	0.520
RLE-illness	0	3	0.37	0.617
RLE-social	0	3	0.41	0.620

Abbreviations: BSI, Brief Symptom Inventory; RLE, recent life stress; RLE-financial, financial difficulties; RLE-illness, illness/injury; RLE-relationship, intimate relationship problems; RLE-social, social network disturbances.

**Table 2:** Main effect and interactions of the *5-HTTLPR* polymorphism on depression symptoms in the combined population sample of Budapest and Manchester

*Combined sample*	*ADD*				*DOM*				*REC*			
*5-HTTLPR polymorphism*	*β*	*s.d.*	P	*FDR* Q	*β*	*s.d.*	P	*FDR* Q	*β*	*s.d.*	P	*FDR* Q
Main effect	0.0099	0.0278	0.7205	0.2522	0.0070	0.0414	0.8674	0.2860	0.0225	0.0504	0.6556^§^	0.2390
Interaction with RLE	0.0446	0.0287	**0.0327**	0.0606	0.0455	0.0306	0.1370	0.1152	0.0798	0.0384	**0.0381**	0.0606
Interaction with RLE-relationship	0.1001	0.0726	0.1684	0.1340	0.1329	0.1074	0.2160	0.1350	0.1341	0.1338	0.3164	0.1579
Interaction with RLE-financial	0.1208	0.0506	***0.0172***^***§***^	0.0375	0.0777	0.0760	0.3067	0.1579	0.2899	0.0927	***0.0018***^§*^	0.0156
Interaction with RLE-illness	0.1070	0.0442	***0.0156***	0.0375	0.1711	0.0653	***0.0088***	0.0349	0.0979	0.0816	0.2304	0.1377
Interaction with RLE-social	−0.0362	0.0444	0.4143	0.1774	−0.0245	0.0641	0.7021	0.2508	−0.0872	0.0843	0.3010	0.1579

Abbreviations: ADD, additive model; DOM, dominant model; FDR, false discovery rate; REC, recessive model; RLE, recent life stress; RLE-financial, financial difficulties; RLE-relationship, intimate relationship problems; RLE-illness, illness/injury; RLE-social, social network disturbances.

Bold type denotes significant (*P<*0.05) values; bold italics indicates significant *P*-values surviving correction for multiple testing (*P<*0.05, FDR *q*<0.05). Effects replicated (signifcant after correction) in the Budapest (§) and Manchester (*) samples are also marked.

**Table 3:** Main effect and interactions of the *5-HTTLPR* polymorphism on depression symptoms in the population sample of Budapest

*Budapest sample*	*ADD*				*DOM*				*REC*			
*5-HTTLPR polymorphism*	*β*	*s.d.*	P	*FDR* Q	*β*	*s.d.*	P	*FDR Q*	*β*	*s.d.*	P	*FDR* Q
Main effect	0.0645	0.0328	**0.0497**	0.0669	0.0445	0.0485	0.3589	0.1653	0.1503	0.0606	***0.0133***	0.0375
Interaction with RLE	0.0457	0.0269	0.0899	0.0968	0.0523	0.0394	0.1850	0.1349	0.0796	0.0504	0.1146	0.1114
Interaction with RLE-relationship	0.0517	0.0857	0.5466	0.2087	0.0183	0.1237	0.8826	0.2860	0.1610	0.1634	0.3248	0.1579
Interaction with RLE-financial	0.1916	0.0701	***0.0070***	0.0349	0.1416	0.1122	0.2074	0.1350	0.4320	0.1263	***0.0007***	0.0115
Interaction with RLE-illness	0.0884	0.0518	0.0887	0.0968	0.1574	0.0751	**0.0365**	0.0606	0.0590	0.1009	0.5587	0.2087
Interaction with RLE-social	−0.0311	0.0534	0.5604	0.2087	0.0120	0.0761	0.8749	0.2860	−0.1259	0.1016	0.2156	0.1350

Abbreviations: ADD, additive model; DOM, dominant model; FDR, false discovery rate; REC, recessive model; RLE, recent life stress; RLE-financial, financial difficulties; RLE-illness, illness/injury; RLE-relationship, intimate relationship problems; RLE-social, social network disturbances.

Bold type denotes significant (*P*<0.05) values; bold italics indicate significant *P*-values surviving correction for multiple testing (*P*<0.05, FDR *q*<0.05).

**Table 4:** Main effect and interactions of the *5-HTTLPR* polymorphism on depression symptoms in the population sample of Manchester

*Manchester sample*	*ADD*				*DOM*				*REC*			
*5-HTTLPR polymorphism*	*β*	*s.d.*	P	*FDR* Q	*β*	*s.d.*	P	*FDR* Q	*β*	*s.d.*	P	*FDR* Q
Main effect	−0.0442	0.0388	0.2538	0.1433	−0.0422	0.0584	0.4701	0.1959	−0.082	0.0694	0.2361	0.1377
Interaction with RLE	0.0470	0.0280	0.0941	0.0968	0.0396	0.0411	0.3354	0.1586	0.0942	0.0513	0.0662	0.0828
Interaction with RLE-relationship	0.1373	0.1018	0.1776	0.1349	0.2303	0.1532	0.1331	0.1152	0.1124	0.1833	0.5398	0.2087
Interaction with RLE-financial	0.1308	0.0661	**0.0480**	0.0669	0.0819	0.0977	0.4024	0.1774	0.3148	0.1220	***0.0010***	0.0349
Interaction with RLE-illness	0.0929	0.0626	0.1382	0.1152	0.1212	0.0941	0.1978	0.1350	0.1240	0.1114	0.2656	0.1453
Interaction with RLE-social	−0.0392	0.0611	0.5214	0.2087	−0.0296	0.0892	0.7403	0.2540	−0.0943	0.1159	0.4157	0.1774

Abbreviations: ADD, additive model; DOM, dominant model; FDR, false discovery rate; REC, recessive model; RLE, recent life stress; RLE-financial, financial difficulties; RLE-illness, illness/injury; RLE-relationship, intimate relationship problems; RLE-social, social network disturbances.

Bold type denotes significant (*P<*0.05) values; bold italics indicates significant *P*-values surviving correction for multiple testing (*P<*0.05, FDR *q*<0.05).
