# Supplementary material for: Financial difficulties but not other types of recent negative life events show strong interactions with 5-HTTLPR genotype in the development of depressive symptoms
Source: Transl Psychiatry. 2016 May 3;6(5):e798–. doi: 10.1038/tp.2016.57 (PMC5070066; doi:10.1038/tp.2016.57)
Supplement: Supplementary Table 1 [file tp201657x1.docx]

**Supplementary Table 1. Pearson’s correlations between the four subscales of Recent Life Events**

|  | | RLE-relationship | RLE-financial | RLE-illness /problems | RLE-social |
| --- | --- | --- | --- | --- | --- |
| RLE-relationship | Correlation coefficient | 1 |  |  |  |
|  | Sig. p-value |  |  |  |  |
|  | | | | | |
| RLE-financial | Correlation coefficient | 0.149 | 1 |  |  |
|  | Sig. p-value | 0.000 |  |  |  |
|  | | | | | |
| RLE-illness | Correlation coefficient | 0.159 | 0.187 | 1 |  |
|  | Sig. p-value | 0.000 | 0.000 |  |  |
|  | | | | | |
| RLE-social | Correlation coefficient | 0.020 | 0.048 | 0.084 | 1 |
|  | Sig. p-value | 0.317 | 0.170 | 0.000 |  |

RLE-relationship: intimate relationship problems; RLE-financial: financial difficulties; RLE-illness: Illness/injury; RLE-social: social network disturbances; sig.: significance.
