# Supplementary material for: Financial difficulties but not other types of recent negative life events show strong interactions with 5-HTTLPR genotype in the development of depressive symptoms
Source: Transl Psychiatry. 2016 May 3;6(5):e798–. doi: 10.1038/tp.2016.57 (PMC5070066; doi:10.1038/tp.2016.57)
Supplement: Supplementary Table 2 [file tp201657x2.docx]

**Supplementary Table 2 Main effect of the *5-HTTLPR* on the occurrence of life stress factors**

|  | Combined | | | Budapest | | | Manchester | | |
| --- | --- | --- | --- | --- | --- | --- | --- | --- | --- |
|  | ADD | DOM | REC | ADD | DOM | REC | ADD | DOM | REC |
| RLE whole | 0.1051 | 0.0647 | 0.4881 | 0.1007 | 0.0850 | 0.3831 | 0.3038 | 0.2212 | 0.6993 |
| RLE-relationship | 0.3991 | 0.4869 | 0.4940 | 0.6041 | 0.9352 | 0.3920 | 0.4460 | 0.3638 | 0.7761 |
| RLE-financial | 0.7742 | 0.3144 | 0.4824 | 0.5429 | 0.7886 | 0.1441 | 0.3354 | 0.2055 | 0.8267 |
| RLE-illness | 0.5200 | 0.4371 | 0.8242 | 0.1534 | 0.1397 | 0.4324 | 0.8221 | 0.9026 | 0.7970 |
| RLE-social | 0.0871 | 0.0856 | 0.3111 | 0.1445 | 0.3155 | 0.1509 | 0.3126 | 0.1579 | 0.8976 |

p values from linear regression analysis are shown
