# Supplementary material for: Financial difficulties but not other types of recent negative life events show strong interactions with 5-HTTLPR genotype in the development of depressive symptoms
Source: Transl Psychiatry. 2016 May 3;6(5):e798–. doi: 10.1038/tp.2016.57 (PMC5070066; doi:10.1038/tp.2016.57)
Supplement: Supplementary Table 3 [file tp201657x3.docx]

**Supplementary Table S3** Items of The List of Life Threatening Experiences questionnaire corresponding to each of the 4 categories used in our analyses. Subjects had to indicate as yes or no if the event happened to them in the previous one year.

| **RLE-relationship** (life events related to intimate relationships including marital difficulties, or break-up of steady relationship) |
| --- |
| You had a separation due to marital difficulties |
| You broke off a steady relationship. |
| **RLE-financial** (financial problem-related life events including being unemployed or seeking work, being fired, or going through a financial crisis) |
| You became unemployed or you were seeking work unsuccessfully for more than one month. |
| You were sacked from your job. |
| You had a major financial crisis. |
| **RLE-illness/problems** (life events related to your illness or injury or severe problems) |
| You yourself suffered a serious illness, injury or an assault. |
| You had a serious problem with a close friend, neighbour or relative. |
| You had problems with the police and a court appearance. |
| Something you valued was lost or stolen. |
| **RLE-social** (problems related to social network, that is serious illness in close relatives or death of close relatives) |
| A serious illness, injury or assault happened to a close relative. |
| Your parent, child, spouse died. |
| A close family friend or another relative (aunt, cousin, grandparent) died. |

Brugha T, Bebbington P, Tennant C, Hurry J. The List of Threatening Experiences: a subset of 12 life event categories with considerable long-term contextual threat. *Psychol Med* 1985; **15**(1)**:** 189-194.
