# Supplementary material for: Financial difficulties but not other types of recent negative life events show strong interactions with 5-HTTLPR genotype in the development of depressive symptoms
Source: Transl Psychiatry. 2016 May 3;6(5):e798–. doi: 10.1038/tp.2016.57 (PMC5070066; doi:10.1038/tp.2016.57)
Supplement: Supplementary Table 4 [file tp201657x4.docx]

**Supplementary Table S4.** Frequency of subjects reporting 0,1,2,3 RLE-financial and 0,1,2,3,4 RLE-illness events

|  | RLE-financial | | RLE-illness | |
| --- | --- | --- | --- | --- |
| Number of events reported | frequency | percent | frequency | percent |
| 0 | 1826 | 81.7 | 1552 | 69.5 |
| 1 | 325 | 14.5 | 557 | 24.9 |
| 2 | 71 | 3.2 | 107 | 4.8 |
| 3 | 12 | 0.5 | 18 | 0.8 |
| 4 |  |  | 0 | 0 |
