# Supplementary material for: Financial difficulties but not other types of recent negative life events show strong interactions with 5-HTTLPR genotype in the development of depressive symptoms
Source: Transl Psychiatry. 2016 May 3;6(5):e798–. doi: 10.1038/tp.2016.57 (PMC5070066; doi:10.1038/tp.2016.57)
Supplement: Supplementary Table 5 [file tp201657x5.docx]

**Supplementary Table S5.** Main effect and interactions with life events of the *5-HTTLPR* polymorphism on depression symptoms in our three samples, among women.

|  |  | **ADD** | | **DOM** | | **REC** | |
| --- | --- | --- | --- | --- | --- | --- | --- |
|  |  | β | P-value | β | P-value | β | P-value |
| Combined sample | Main effect | 0.0055 | 0.8751 | -0.0100 | 0.8456 | 0.0331 | 0.6001 |
|  | Interaction with RLE | 0.0180 | 0.4883 | 0.0355 | 0.3428 | 0.0047 | 0.9225 |
|  | Interaction with RLE-relationship | 0.0131 | 0.8830 | 0.0676 | 0.6131 | -0.0541 | 0.7364 |
|  | Interaction with RLE-financial | 0.0566 | 0.3702 | 0.0620 | 0.5132 | 0.0992 | 0.3937 |
|  | Interaction with RLE-illness | 0.0650 | 0.2393 | 0.1332 | *0.0955* | 0.0075 | 0.9426 |
|  | Interaction with RLE-social | -0.0549 | 0.3083 | -0.0238 | 0.7573 | -0.1571 | 0.1313 |
| Budapest sample | Main effect | 0.0897 | **0.0232** | 0.0712 | 0.2160 | 0.1930 | **0.0085** |
|  | Interaction with RLE | 0.0081 | 0.7991 | 0.0541 | 0.2445 | -0.0575 | 0.3527 |
|  | Interaction with RLE-relationship | -0.0964 | 0.3710 | -0.0713 | 0.6558 | -0.2165 | 0.2723 |
|  | Interaction with RLE-financial | 0.1021 | 0.2384 | 0.1824 | 0.2075 | 0.1288 | 0.4075 |
|  | Interaction with RLE-illness | 0.0375 | 0.5496 | 0.1073 | 0.2263 | -0.0523 | 0.6769 |
|  | Interaction with RLE-social | -0.0861 | 0.1569 | -0.0276 | 0.7510 | -0.2556 | **0.0284** |
| Manchester sample | Main effect | -0.0732 | 0.1308 | -0.0933 | 0.2013 | -0.1035 | 0.2340 |
|  | Interaction with RLE | 0.0257 | 0.4639 | 0.0198 | 0.6957 | 0.0549 | 0.3966 |
|  | Interaction with RLE-relationship | 0.1065 | 0.3779 | 0.1814 | 0.3199 | 0.0862 | 0.6892 |
|  | Interaction with RLE-financial | 0.0987 | 0.2339 | 0.0702 | 0.5622 | 0.2241 | 0.1469 |
|  | Interaction with RLE-illness | 0.0608 | 0.4346 | 0.0952 | 0.4070 | 0.0580 | 0.6810 |
|  | Interaction with RLE-social | -0.0390 | 0.6034 | -0.0039 | 0.9710 | -0.1427 | 0.3280 |

RLE-relationship: intimate relationship problems; RLE-financial: financial difficulties; RLE-illness: Illness/injury; RLE-social: social network disturbances; ADD: additive model, DOM: dominant model, REC: recessive model. **Bold** type denotes significant (p<0.05) values; *italics* indicate trends.
