# Supplementary material for: Financial difficulties but not other types of recent negative life events show strong interactions with 5-HTTLPR genotype in the development of depressive symptoms
Source: Transl Psychiatry. 2016 May 3;6(5):e798–. doi: 10.1038/tp.2016.57 (PMC5070066; doi:10.1038/tp.2016.57)
Supplement: Supplementary Table 6 [file tp201657x6.docx]

**Supplementary Table S6.** Main effect and interactions with life events of the *5-HTTLPR* polymorphism on depression symptoms in our three samples, among men.

|  |  | **ADD** | | **DOM** | | **REC** | |
| --- | --- | --- | --- | --- | --- | --- | --- |
|  |  | β | P-value | β | P-value | β | P-value |
| Combined sample | Main effect | 0.0194 | 0.6677 | 0.0471 | 0.4881 | -0.0046 | 0.9551 |
|  | Interaction with RLE | 0.1112 | **0.0014^§*^** | 0.0810 | 0.1290 | 0.2364 | **0.0001^§*^** |
|  | Interaction with RLE-relationship | 0.2880 | **0.0201^§^** | 0.2575 | 0.1467 | 0.6117 | **0.0116^§^** |
|  | Interaction with RLE-financial | 0.2725 | **0.0010^§*^** | 0.1130 | 0.3609 | 0.7257 | **<0.0001^§*^** |
|  | Interaction with RLE-illness | 0.1972 | **0.0066^§^** | 0.2783 | **0.0142** | 0.2586 | **0.0418** |
|  | Interaction with RLE-social | 0.0255 | 0.7449 | -0.0080 | 0.9454 | 0.0971 | 0.4921 |
| Budapest sample | Main effect | 0.0015 | 0.9798 | -0.0312 | 0.7290 | 0.0503 | 0.6429 |
|  | Interaction with RLE | 0.1254 | **0.0151** | 0.0212 | 0.7868 | 0.3347 | **0.0001** |
|  | Interaction with RLE-relationship | 0.2947 | **0.0411** | 0.1567 | 0.4342 | 0.9056 | **0.0020** |
|  | Interaction with RLE-financial | 0.3977 | **0.0013** | 0.0742 | 0.6810 | 1.2130 | **<0.0001** |
|  | Interaction with RLE-illness | 0.1882 | **0.0471** | 0.2658 | *0.0657* | 0.2484 | 0.1518 |
|  | Interaction with RLE-social | 0.1138 | 0.3006 | 0.0977 | 0.5351 | 0.2280 | 0.2644 |
| Manchester sample | Main effect | 0.0255 | 0.6855 | 0.0803 | 0.3957 | -0.0323 | 0.7742 |
|  | Interaction with RLE | 0.1057 | **0.0201** | 0.1251 | *0.0752* | 0.1747 | **0.0322** |
|  | Interaction with RLE-relationship | 0.2546 | 0.1887 | 0.4504 | 0.1183 | 0.1800 | 0.6204 |
|  | Interaction with RLE-financial | 0.2178 | **0.0418** | 0.1384 | 0.3948 | 0.5132 | **0.0076** |
|  | Interaction with RLE-illness | 0.1567 | 0.1285 | 0.2178 | 0.1849 | 0.2217 | 0.2033 |
|  | Interaction with RLE-social | -0.0132 | 0.8996 | -0.0587 | 0.7132 | 0.0436 | 0.8154 |

RLE-relationship: intimate relationship problems; RLE-financial: financial difficulties; RLE-illness: Illness/injury; RLE-social: social network disturbances; ADD: additive model, DOM: dominant model, REC: recessive model. **Bold** type denotes significant (p<0.05) values; *italics* indicate trends. In case of the combined sample, effects also significant in the Budapest (§) and Manchester (*) samples are also marked.
